# Supplementary material for: Association of body index with fecal microbiome in children cohorts with ethnic–geographic factor interaction: accurately using a Bayesian zero-inflated negative binomial regression model
Source: mSystems. 2024 Nov 21;9(12):e01345-24. doi: 10.1128/msystems.01345-24 (PMC11651110; doi:10.1128/msystems.01345-24)
Supplement: Supplemental material — Appendix S1 and Fig. S1 to S5. [file msystems.01345-24-s0001.docx]

**Association of Body Index with Fecal Microbiome in Children Cohorts with Ethnic-Geographic Factor Interaction: Accurately Using a Bayesian Zero-inflated Negative Binomial Regression Model**

Jian Huang^1,2^, Yanzhuan Lu^1,2^, Fengwei Tian^3,4^, and Yongqing Ni^1,2*^

^1^School of Food Science and Technology, Shihezi University, Shihezi, Xinjiang, China

^2^Key Laboratory of Xinjiang Special Probiotics and Dairy Technology, Shihezi University, Shihezi, Xinjiang, China

^3^State Key Laboratory of Food Science and Resources, Jiangnan University, Wuxi, Jiangsu, China

^4^School of Food Science and Technology, Jiangnan University, Wuxi, Jiangsu, China

*Shihezi University, Shihezi, Xinjiang, China, niyqlzu@sina.com

**Appendix S1 Supplementary algorithms. estimate zero-inflated probability and** $\boldsymbol{\phi}$ **parameter.**

For the bacteria count data, we estimate the data properties of each ASV/OTU to determine whether it conforms to the zero-infa negative binomial distribution.

It is vitally important for us to choose the right model.

According to the Formula (1) of the text part, the variance and mean are knowable.

$\mu_{nb}=\frac{\mu}{1-\phi}$ （1）

At this point, the mean and variance in the negative binomial distribution are:

$$Var_{nb}=\frac{\left( \mu+\frac{\mu^{2}}{\theta}+\mu^{2} \right)\left( 1-\phi\right)-\mu^{2}}{\left( 1-\phi\right)^{2}}$$

By utilizing the variance and mean, we can determine the dispersion parameters $\theta$ of y.

$$\theta_{nb}=\frac{\theta}{1-\phi\theta-\phi}$$

In this way, we can calculate the true probability of zero-inflation:

$$\frac{\mathrm{zerocount}}{n} = \phi+ \left( 1-\phi\right){\frac{\theta\left( 1-\phi\right)}{\theta\left( 1-\phi\right)+\mu\left( 1-\theta\phi-\phi\right)}}^{\left( \frac{\theta}{1-\phi-\theta\phi} \right)}$$

According to the above equation, we can solve the $\phi$ parameter.


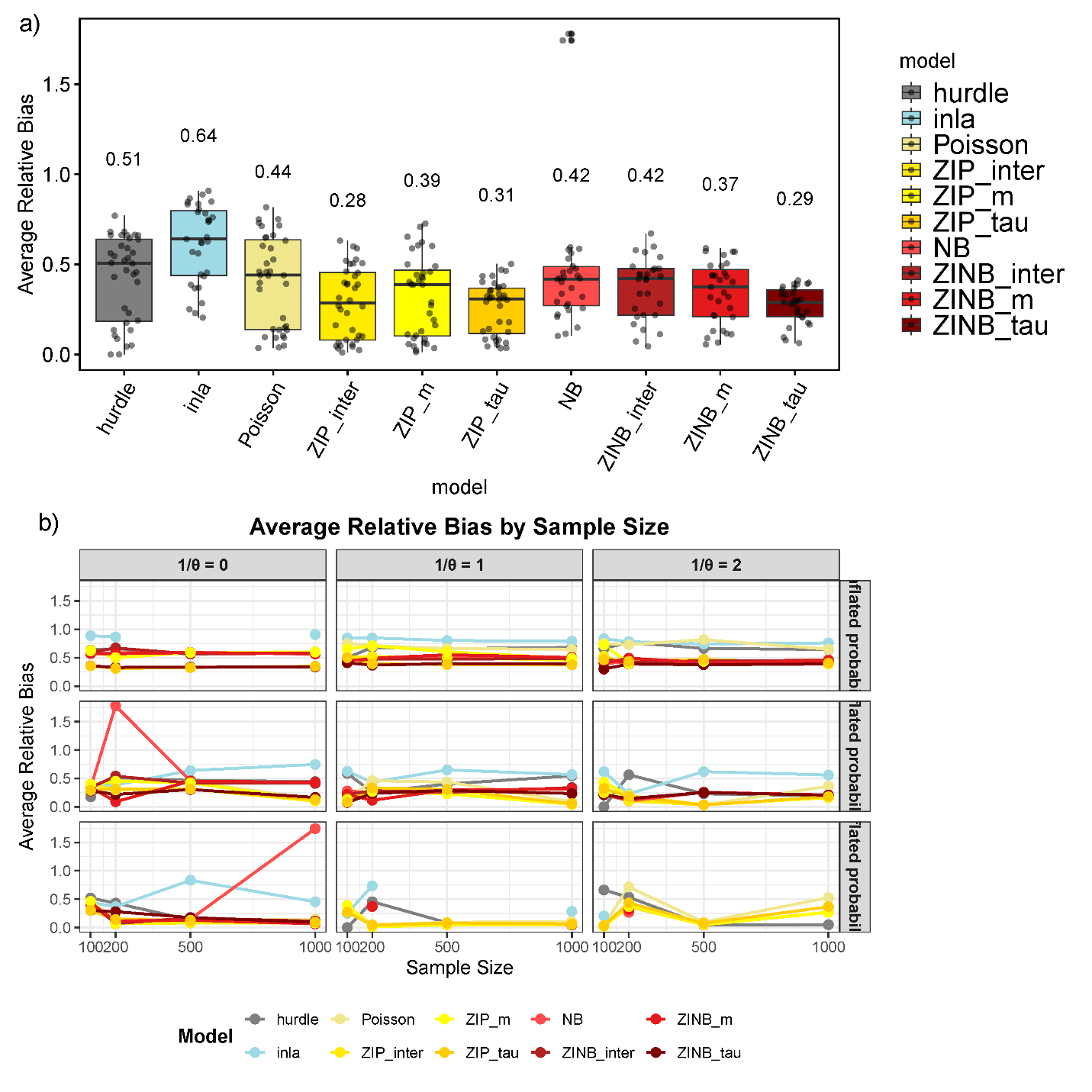


**Figure S1.** Fits of ten models in simulation experiment of bacterial counts when covariate coefficient = 0.2. a) The box plot illustrates the fitted ARB values for the ten models across all scenarios, with distinct colors representing different models-red for ZINB models and yellow for ZIP models. The value displayed above each box plot denotes the median ARB for each model. b) The fitting accuracy of 10 models in different cases, where each row corresponds to a distinct level of dispersion (null 1/θ =0, median 1/θ =1 and high 1/θ = 2) and each column represents a different probability of zero-inflation (null 0, median 0.2 and high 0.5).

**
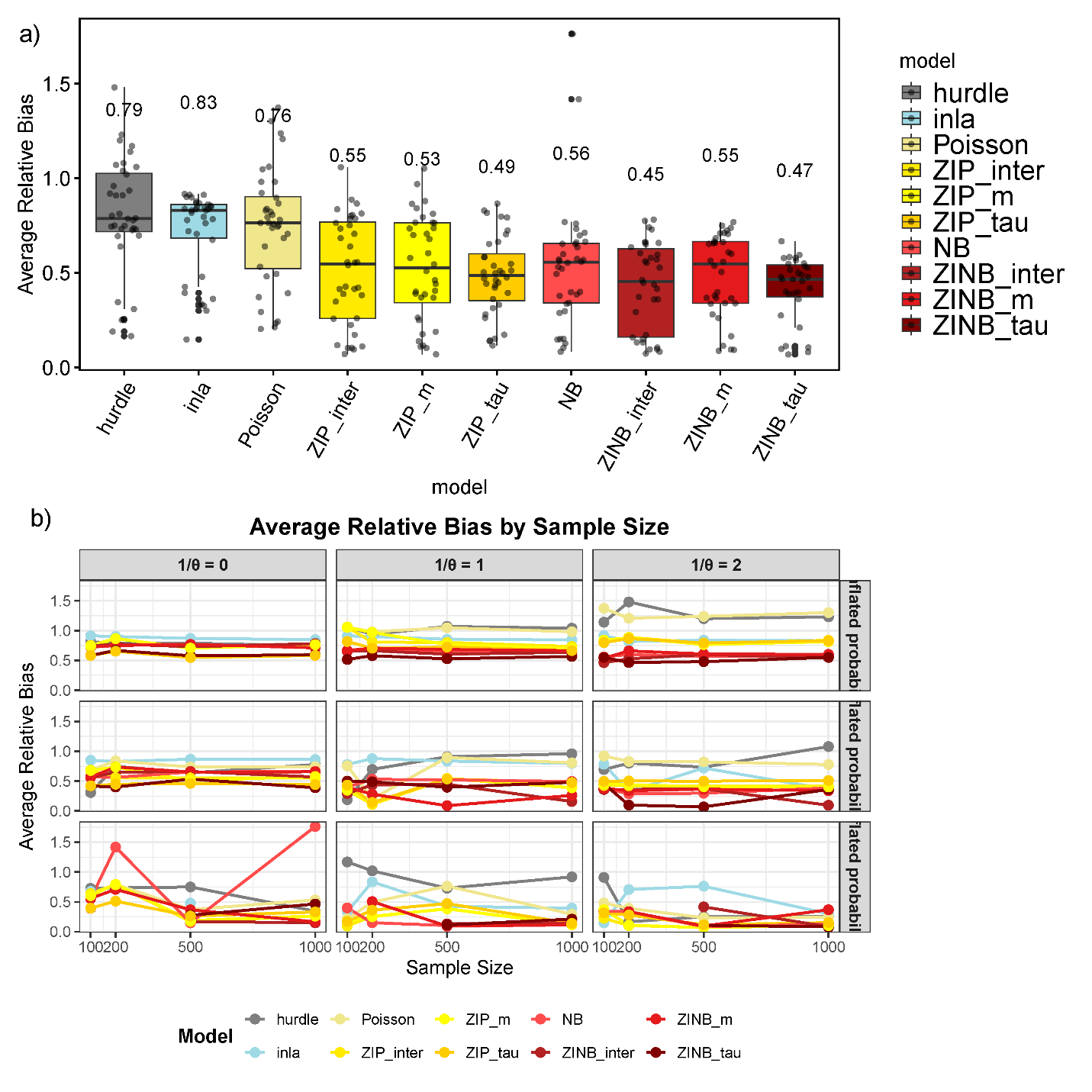
Figure S2.** Fits of ten models in simulation experiment of bacterial counts when covariate coefficient = 0.5. a) The box plot illustrates the fitted ARB values for the ten models across all scenarios, with distinct colors representing different models-red for ZINB models and yellow for ZIP models. The value displayed above each box plot denotes the median ARB for each model. b) The fitting accuracy of 10 models in different cases, where each row corresponds to a distinct level of dispersion (null 1/θ =0, median 1/θ =1 and high 1/θ = 2) and each column represents a different probability of zero-inflation (null 0, median 0.2 and high 0.5).

**
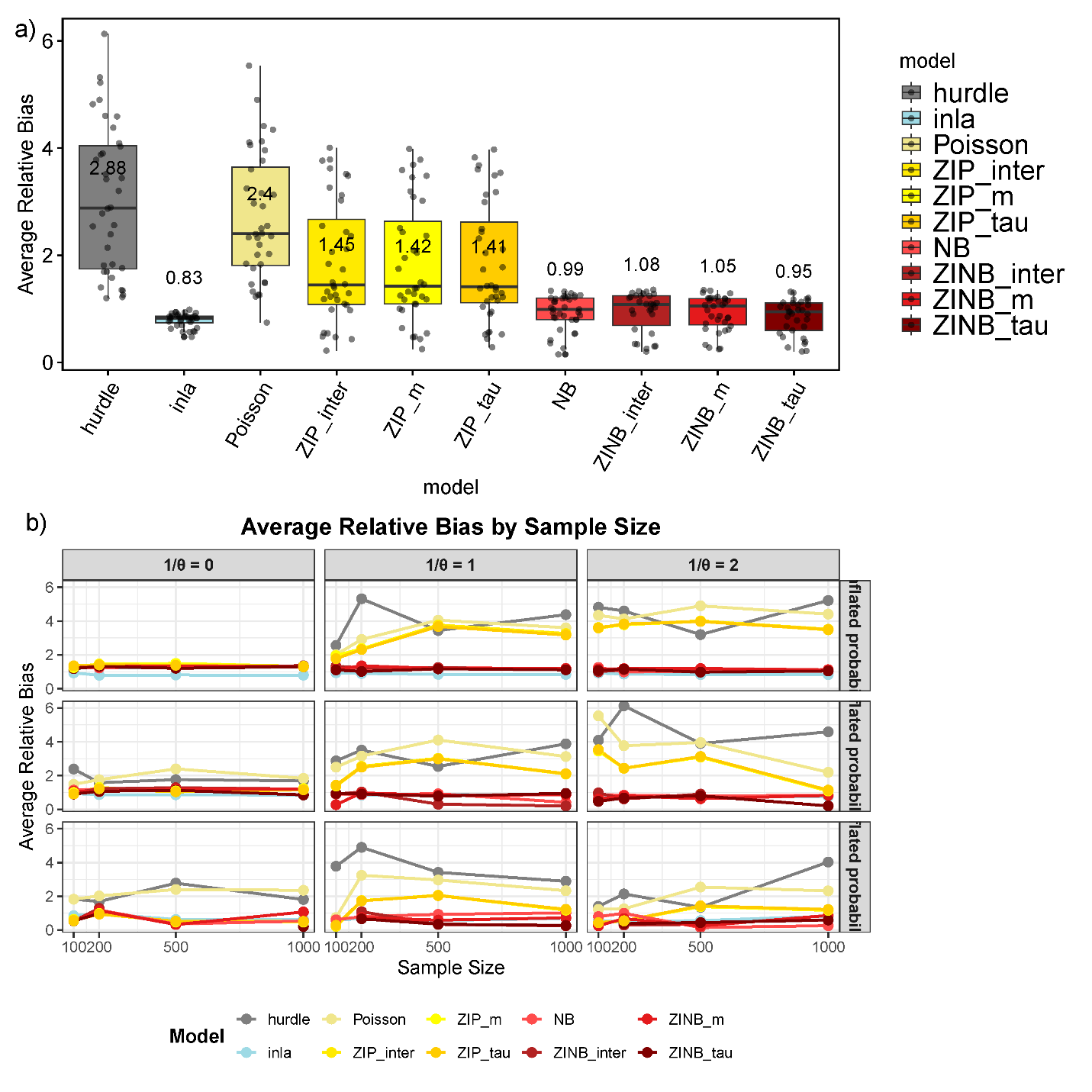
Figure S3.** Fits of ten models in simulation experiment of bacterial counts when covariate coefficient = 1.0. a) The box plot illustrates the fitted ARB values for the ten models across all scenarios, with distinct colors representing different models-red for ZINB models and yellow for ZIP models. The value displayed above each box plot denotes the median ARB for each model. b) The fitting accuracy of 10 models in different cases, where each row corresponds to a distinct level of dispersion (null 1/θ =0, median 1/θ =1 and high 1/θ = 2) and each column represents a different probability of zero-inflation (null 0, median 0.2 and high 0.5).


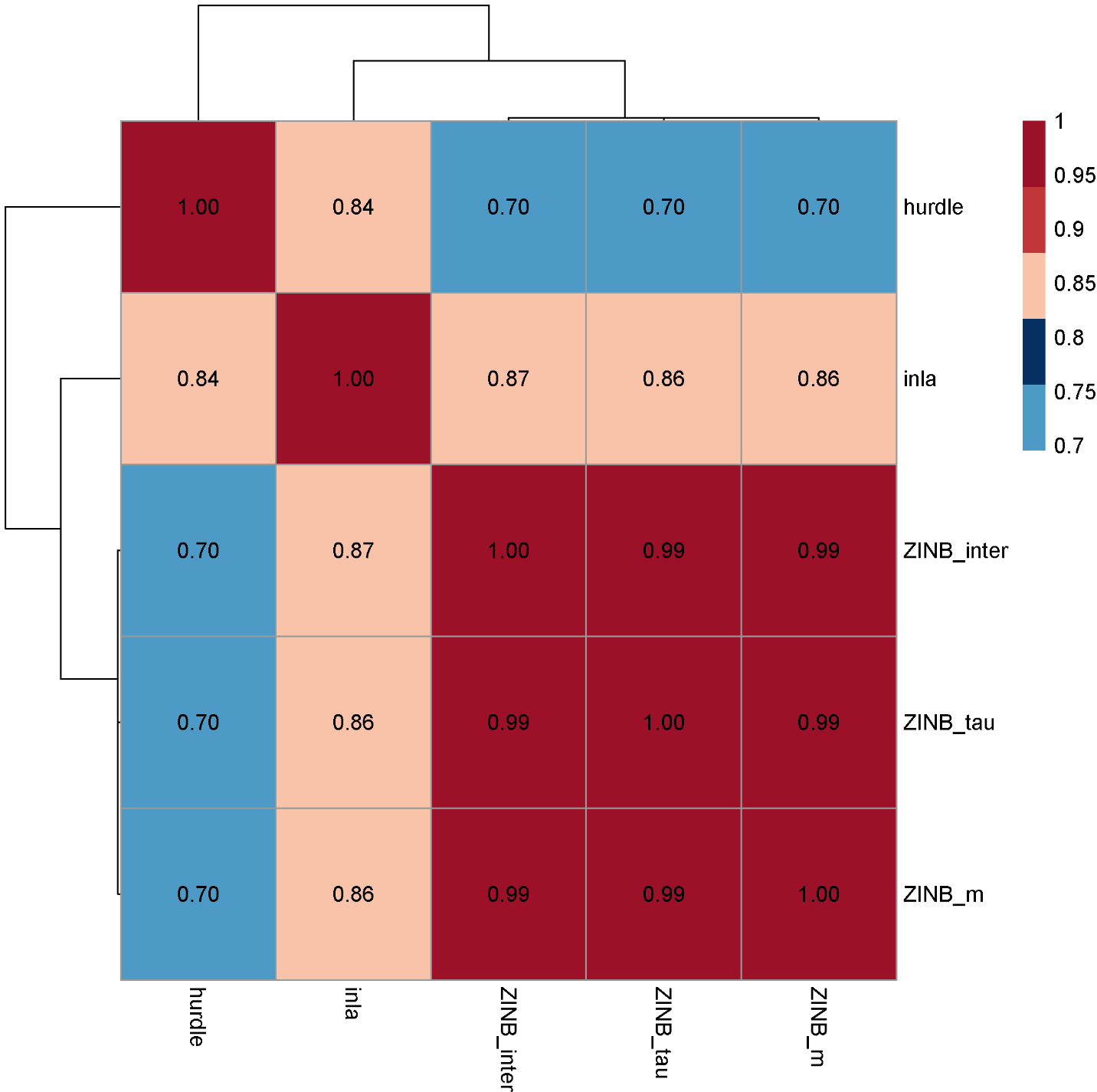


**Figure S4.** Heatmap of comparing different model results fitting of age covariates. The Spearman correlation coefficient of each model was calculated by comparing the correlation coefficient of each ASV to age predicted by 5 models.


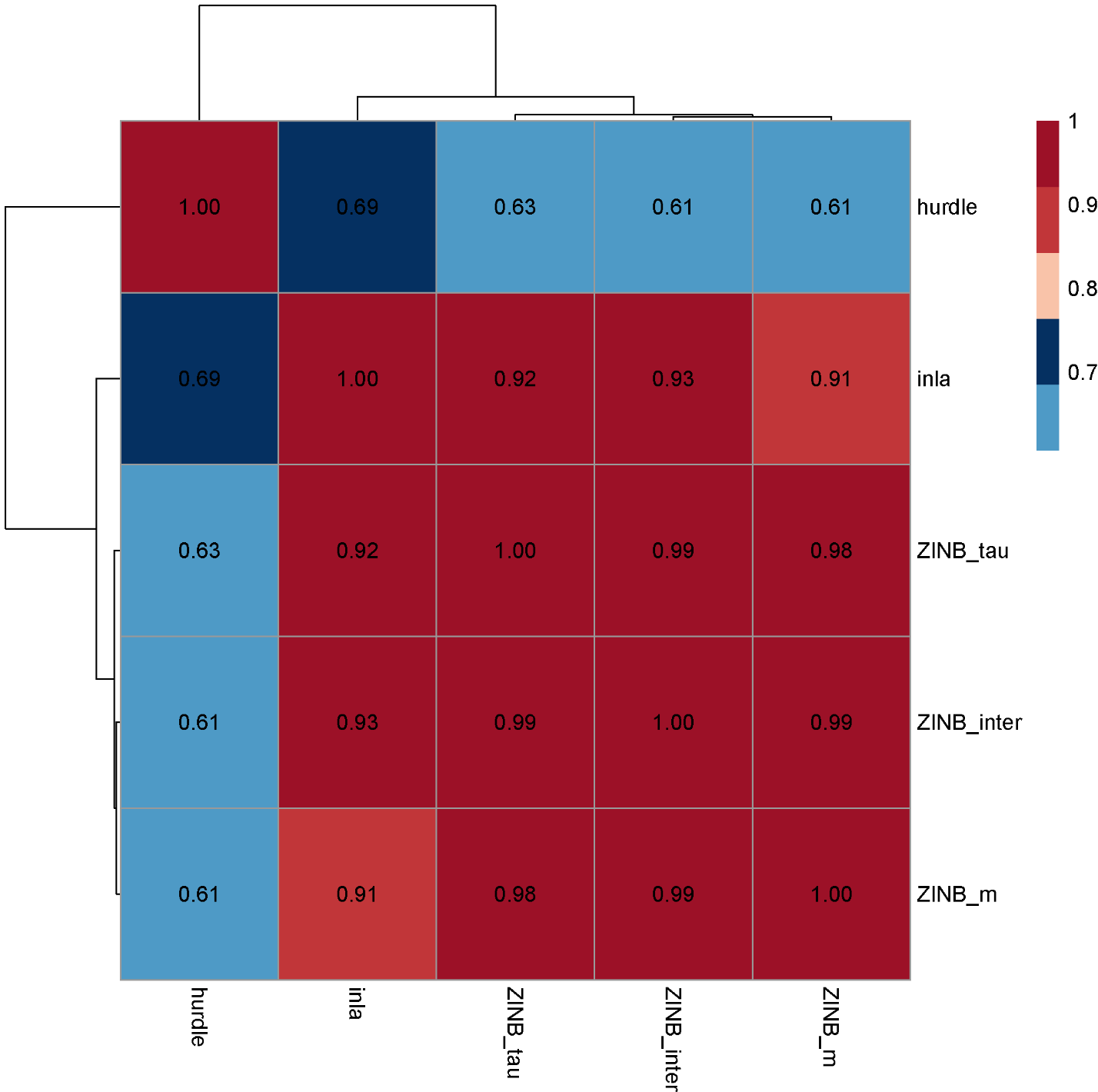


**Figure S5.** Heatmap of comparing different model results fitting of BMI-for-Age Zscores covariates. The Spearman correlation coefficient of each model was calculated by comparing the correlation coefficient of each ASV to age predicted by 5 models.
